# 

**Published:** 1892-07-30

**Authors:** 


### CONTENTS.

**The Crisis of the Voluntary Hospital** . . . . . . 289
**Passing Topics.**—Street Collections . . . . . 290
**Medical History from the Earliest Times.** By E. T. WITHINGTON, M.A., M.B.Oxon. XIX.—Celsus and Ancient Surgery . . . . . . . . . . 291
**Everybody's Page** . . . . . . . . . . 292
**The Attitude of Great Writers towards Disease.** VII.—Dante . . . . . . . . . . 293
**Faith Healing** . . . . . . . . . . 294
**Annotations.**—Health and Beauty—"Below Par"—A Fossil Institution . . . . . . . . . . 295
**Notes and News** . . . . . . . . . . 296
**Around the Hospitals** . . . . . . . . . . 297
**Scraps and Gleanings** . . . . . . . . . . 297
**The Practitioners' Mirror and Retrospect—**
MEDICINE.—Paracentesis Pericardii . . . . . . 298
SURGERY.—Conditions Simulating Renal Calculus II. . . 298
THERAPEUTICS.—Adonis Vernalis . . . . . . 299
**Editor's Letter Box** . . . . . . . . . . 300
**The Institutional Workshop—**
INSTITUTION LITERATURE.—Asylums of the World. IV.—The Construction of Asylums . . . . . . 301
WITHIN THE HOSPITALS. VII.—The Convalescent Home at Bonchurch . . . . . . . . . . 302
**The London Hospitals.**—Proposed Central Board . . 302
**The Royal Hospital for Incurables, Putney** . . . 302
**The Student of Medicine.** — Appointments—Vacancies—Pass Lists—Athletics.

### EXTRA SUPPLEMENT.—THE NURSING MIRROR.

**En Passant.**—Registration of Nurses—Some of the Effects of Cleaning—Belsey v. McLelland—Royal British Nurses' Association—Death at Lambeth . . . . . . cxxi

**Ventilation, Disinfection, and Diet.** By P. CALDWELL SMITH, M.D. XVI.—Diet and Dietaries—(*concluded*) . . cxxii

**Probationers** . . . . . . . . . . cxxiii

**Our American Sisters** . . . . . . . . . . cxxiv

**Nurses' Bookshelf** . . . . . . . . . . cxxiv

**For Reading to the Sick.**—Mists . . . . . . cxxv

**The Nurses' Journal Club**—Johns Hopkins Hospital, Baltimore, U.S. . . . . . . . . . . cxxv

**Everybody's Opinion.**—Re Longton Cottage Hospital—The History of Registration—Nursing in Asylums—Trained Male Nurses . . . . . . . . . . cxxvi

**The Hamilton Association—Notes and Queries** . . cxxvii

**The Real Mrs. Skeffington** . . . . . . cxxviii

